# Copland’s Dictionary of Practical Medicine

**Published:** 1845-07

**Authors:** 


*Copland's Dictionary of Practical Medicine.*

Some months ago, we noticed the intended re-publication of Dr. Copland's Dictionary of Practical Medicine, by the Messrs.

Harpers of New York, to be edited by Professor Lee, and the successive appearance of the *four* first numbers. Not having received any numbers since then, we have made inquiry, and find that the work has progressed as far as the publication of No. IX. Of the subjects embraced in these later numbers, and the amount of the editor's additions, we are of course unable to speak.

